# Comparison of Chondrocyte Behaviors Between Silk Microfibers and Polycaprolactone Microfibers in Tissue Engineering and Regenerative Medicine Applications

**DOI:** 10.3390/bioengineering11121209

**Published:** 2024-11-29

**Authors:** Guang-Zhen Jin

**Affiliations:** Institute of Tissue Regeneration Engineering, Dankook University, Cheonan 31116, Republic of Korea; gzhjin@dankook.ac.kr; Tel.: +82-41-550-3082-4

**Keywords:** chondrogenic phenotype, microfibers, proliferation, polycaprolactone, silk

## Abstract

Silk and polycaprolactone (PCL), derived from natural and synthetic sources, respectively, are suture materials commonly used in surgery. Beyond their application in sutures, they are also compelling subjects in regenerative medicine and tissue engineering. This study evaluated the effects of degummed silk microfibers compared to electrospun PCL microfibers of a similar diameter on chondrocyte behavior. The two types of microfibers were analyzed using scanning electron microscopy (SEM), real-time PCR, Western blotting, and DMMB analysis. The results demonstrated that the silk microfibers exhibited a higher proliferative cell rate over time compared to the PCL microfibers. Additionally, the expression of chondrogenic phenotypes was significantly upregulated, while the marker for hypertrophic chondrocytes—type X collagen—was downregulated in cell-laden silk microfibers compared to cell-laden PCL microfibers. These findings suggest that natural degummed silk microfibers may be a viable option for repairing damaged cartilage in the future of orthopedic surgery and bioengineering.

## 1. Introduction

Surgical sutures for wound closure are categorized into natural and synthetic types. Natural sutures consist of materials like silk, cotton, and catgut, whereas synthetic options are derived from polymers such as polycaprolactone (PCL), poly(glycolic acid) (PGA), and nylon [1]. These sutures are extensively utilized in clinical surgery, significantly contributing to efficient wound healing.

Silk sutures have been considered generally safe for use in the human body for the past 100 years. They are manufactured from silk fibroin extracted from the cocoons of Bombyx mori silkworms. Silk fibers are protein fibers characterized by a core–shell structure, consisting of a fibroin layer at the core and a sericin layer on the outside. Fibroin makes up 70 to 80% of silk proteins and is known for its excellent biocompatibility, plasticity, and tunable degradation rate, while sericin accounts for about 20–30% of the silk protein content [2]. However, the presence of sericin can trigger an immune response; therefore, it is removed from silk sutures to prevent adverse reactions [3]. Fibroin is an attractive scaffolding biomaterial for regenerative medicine, particularly in cartilage tissue engineering. Most fibroin-based scaffolds are made from regenerated silk fibroin. A study by Agrawal and Pramanik demonstrated that composite scaffolds incorporating regenerated silk fibroin enhance mesenchymal stem cell (MSC) chondrogenic differentiation in dynamic cultures [4]. Research conducted by Yodmuang et al. further supports these findings. They demonstrated that silk-based hydrogels possess comparable mechanical and functional characteristics for cartilage tissue repair [5]. However, to date, no studies have investigated the potential application of natural silk fibroin scaffolds in cartilage tissue engineering.

PCL, a synthetic polymer and a prominent member of the aliphatic polyester family, is widely utilized as a biomaterial in surgical sutures and tissue engineering scaffolds. Pure PCL sutures have a long degradation time, lasting up to two years, which is why their combination with PGA is often employed to accelerate the biodegradation rate [6]. PCL scaffolds are widely utilized in cartilage tissue engineering. Park et al. demonstrated the construction of tissue tracheas by plating human chondrocytes on 3D-printed PCL/gelatin scaffolds and treating them with transforming growth factor-β1 (TGF-β1) [7]. Similarly, Li et al. successfully repaired meniscus defects in rabbits using 3D-printed PCL/silk fibroin biomimetic scaffolds in combination with synovium-derived MSCs [8].

Surgical sutures are also utilized for the delivery of drugs and cells. Preventing surgical site infections is crucial for reducing postoperative morbidity and mortality, thereby ensuring successful surgical outcomes. Antibiotic-coated sutures have proven effective in preventing these infections. Recently, Chen et al. developed a novel braided silk suture that effectively kills bacteria through a combined coating process involving levofloxacin hydrochloride and PCL [9]. Sutures coated with growth factors like the basic fibroblast growth factor and the vascular endothelial growth factor have been demonstrated to improve angiogenesis and facilitate tendon repair in wound healing models [10,11]. Furthermore, sutures embedded with mesenchymal stem cells have improved healing at the surgical site by promoting cell proliferation and differentiation [12,13]. Sutures are a significant focus of research in tissue engineering and regenerative medicine.

This study compared chondrocyte behavior on degummed silk microfibers and electrospun PCL microfibers of comparable fiber diameters. The results indicated that silk microfibers enhanced chondrocyte proliferation and preserved their chondrogenic phenotype more effectively than PCL microfibers, highlighting the superiority of silk microfibers for cartilage repair applications.

## 2. Materials and Methods

### 2.1. Microfiber Preparation

Natural degummed silk microfibers were prepared from the cocoons of Bombyx mori. After boiling for 30 min in a 0.02M Na_2_CO_3_ solution, the cocoon was degummed. Then, the microfibers were washed in deionized water and air-dried overnight on a clean bench.

PCL microfibers (MW = 80,000, Sigma-Aldrich) were produced via electrospinning within a metal-framed 3D plastic box measuring 80 × 70 × 60 cm. Then, 10% (*w*/*v*) PCL was dissolved in chloroform. Electrospinning was performed using a feeding rate of 10 mL/h, a working distance of ~35 cm, and a voltage of 22 kV by the rotation of airflow exerted by an external fan (air speed ~2.5 mph).

Microfiber and cell-containing microfiber morphologies were analyzed using a Hitachi S-3000H (Hitachi S-3000H, Hitachi, Ltd., Tokyo, Japan) scanning electron microscope at 10 kV. From the images obtained, the size distribution of the microfibers was determined.

### 2.2. Culture and Seeding of Cells

The rat chondrocytes used in this study were cryopreserved samples from two passages from a previous study [14]. To prevent the cells from adhering to the bottom of the culture dish during seeding and enhance their attachment to the microfibers, a thin layer of agarose gel was evenly coated on the bottom of the dish. Initially, 10 mg of each type of microfiber was weighed and pre-wetted to promote cell attachment. The specific procedure involved placing the microfibers in a culture dish, completely submerging them in culture medium containing Dulbecco’s Modified Eagle Medium (DMEM, 4.5 g/L glucose), 10% fetal bovine serum, and 1% penicillin/streptomycin (all from Gibco-BRL, Grand Island, NY, USA), and incubating them in an incubator for 30 min. After 30 min, the residual culture medium in the microfibers was gently removed. To estimate the initial volume of culture medium needed for preparing the cell suspension, the culture medium was slowly added to the pre-wetted microfibers using a pipette, ensuring no overflow. Subsequently, 8 × 10^5^ cells were suspended in the estimated volume of culture medium and carefully seeded onto each microfiber scaffold. After seeding, no additional medium was added immediately. The scaffolds were then placed in the incubator for approximately 1 h to allow the cells to fully attach to the scaffolds. Finally, a culture medium containing 50 μg/mL ascorbic acid, 1% insulin–transferrin–selenium (ITS), 0.1 M dexamethasone, and 10 ng/mL TGF-β1 was slowly added. The scaffolds were then returned to the incubator and cultured under a 5% CO_2_ humidified atmosphere for further cultivation.

### 2.3. Cell Growth Assay

Microfibers containing cells were cultured for 1, 4, and 7 days in growth medium. Under an inverted fluorescence microscope (DP2-BSW, Olympus, Tokyo, Japan), cell adhesion and growth were evaluated with Alexa Fluor 488 phalloidin staining (Invitrogen, USA). Cell proliferation was evaluated using the CellTiter 96 Aqueous One Solution Assay (MTS; Promega, Madison, WI, USA). Sample absorbance was recorded at 490 nm with a Molecular Devices microplate reader (Thermo Fisher Scientific, USA). Three replicates were conducted at each time point. The cell proliferative rate was determined using the following formula:

Proliferative rate (%) = OD490 (Day t)/OD490 (Day 1) × 100%.

where OD490 (Day 1) and OD490 (Day t) are the optical density values at the 490 nm wavelength on Day 1 and OD490 (Day t), respectively.

### 2.4. Western Blotting

For the analysis of cell attachment, the cell-laden microfibers were cultured in growth medium and collected after 24 h. We cultured microfibers in chondrogenic medium and measured type II collagen after 14 days of incubation to quantify cartilage-specific marker proteins. The protein concentrations in the supernatants were determined using a Bradford assay (Bio-Rad, USA) following sample lysis in RIPA buffer and centrifugation at 12,000 rpm for 10 min at 4 °C. A 25 μg protein sample was separated using 10% SDS-PAGE and then electrophoretically transferred to PVDF membranes. To prevent non-specific binding, the membrane was incubated in 5% non-fat dry milk for 1 h at room temperature. The membranes were incubated overnight at 4 °C with primary antibodies from Santa Cruz Biotechnology, USA, targeting fibronectin (sc-69681), focal adhesion kinase (FAK, sc-271195), vinculin (sc-55465), paxillin (sc-365379), type II collagen (sc-52658) (all at a 1:200 dilution), and glyceraldehyde-3-phosphate dehydrogenase (GAPDH, 1:1000, sc-32233). The membranes were incubated at room temperature for 2 h with horseradish peroxidase-conjugated anti-mouse or anti-rabbit IgG (1:5000; Santa Cruz Biotechnology, USA). The blots were analyzed using enhanced chemiluminescence (Thermo Scientific, USA) and visualized with the ImageQuant LAS 4000 mini (GE Healthcare Life Sciences, USA). ImageJ software (version 1.8.0, NIH, Bethesda, MD, USA) was used to analyze the bands.

### 2.5. Quantitative Real-Time PCR (qRT-PCR)

Samples were collected from cell-laden microfibers after 14 days of culture in chondrogenic medium. Chondrocyte-related genes were analyzed using qRT-PCR following RNA extraction with TRIzol reagent (Invitrogen). cDNA was synthesized from 1 μg of RNA using the SuperScript first-strand synthesis system (Invitrogen, USA) following the manufacturer’s instructions. SYBR GreenER qPCR SuperMix reagents (Invitrogen, USA) were used for the qRT-PCR. Transcript levels were quantified using the ΔΔCt method, using GAPDH as a reference gene. Table 1 provides the primer sequences for the genes analyzed in qRT-PCR.

### 2.6. Dimethyl Methylene Blue (DMMB) Assay

After 14 days of culture in chondrogenic medium, the GAG content in cell-laden microfibers was evaluated. Samples were digested for 1 h at 60 °C using a 20 mM phosphate-buffered saline solution (pH 6.8) with 300 μg/mL papain. The GAG content was measured at 525 nm using the DMMB assay (Astartebio Ltd., Cat# 8000, USA). A standard curve for GAG quantification was developed using chondroitin sulfate from bovine trachea (Astartebio Ltd., USA). The GAG values were normalized to the total DNA content of the samples, which was determined using the PicoGreen quantitation assay kit (Molecular Probes, USA).

### 2.7. Statistical Analysis

The data were analyzed using Student’s *t*-tests to determine significance. A significance threshold of *p* < 0.05 was employed in our analysis.

## 3. Results

### 3.1. Morphology and Characteristic of Both Microfibers

**Figure 1:**
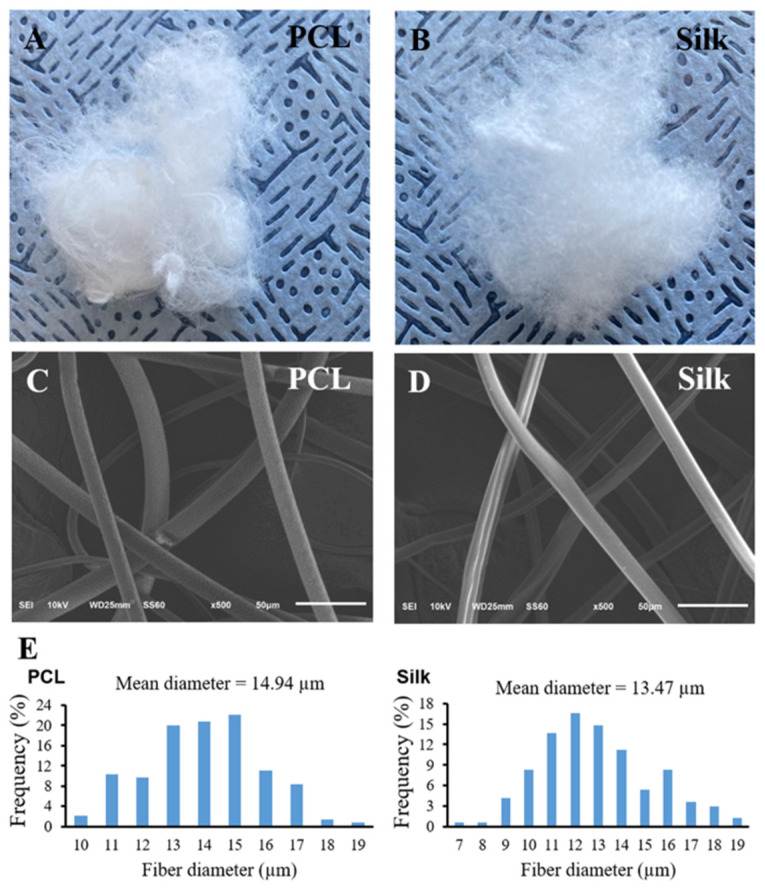
The gross appearance of the natural silk and PCL microfibers (**A**,**B**). SEM morphology of both microfibers (**C**,**D**). Diameter size distribution of both microfibers (**E**).

Figure 1A,B illustrate the gross morphology of both silk and electrospun PCL microfibers. Both microfibers have a similar wool-like appearance. The SEM morphologies of both the silk and PCL microfibers are shown in Figure 1C,D. Most silk microfibers have a smooth and somewhat grooved surface. The smooth surface of the PCL microfibers is due to having been obtained by blowing and spinning the solution. The average diameters of the silk and PCL microfibers are very similar, measuring (13.47 ± 3.09) μm and (14.94 ± 3.81) μm, respectively (Figure 1E).

### 3.2. Cell Adhesion-Related Protein Expression

**Figure 2:**
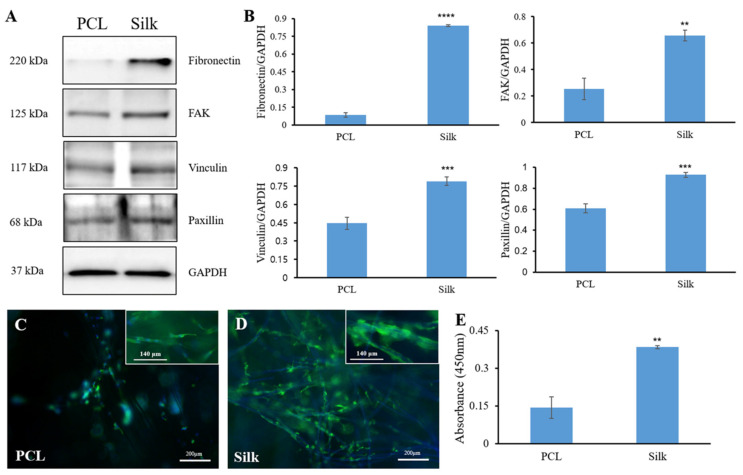
Cell adhesion behaviors on both the silk and PCL microfibers after 24 h of culture. Representative Western blot images of cell adhesion proteins and the expressions of cell adhesion proteins were normalized to GAPDH (**A**,**B**). Significance levels are indicated as follows: ** *p* < 0.01, *** *p* < 0.001, and **** *p* < 0.0001, in comparison to PCL. Fluorescence images of cell adhesion (**C**,**D**). Insets show higher-magnification images. Cell adhesion quantified by MTS assay (**E**). ** *p* < 0.01 vs. PCL.

To compare the cell attachment potential of both microfibers, we investigated the protein expression associated with focal adhesion after 24 h of culture in the growth medium (Figure 2A,B). The Western blotting analysis revealed that fibronectin, an extracellular matrix protein crucial for cell adhesion, exhibited higher expression in silk microfibers than in PCL microfibers. The expressions of focal adhesion proteins such as FAK, vinculin, and paxillin were evaluated. The findings indicated that silk microfibers exhibited significantly higher protein expression levels of FAK, vinculin, and paxillin compared to the PCL microfibers. At the same time, the behavior of cell adhesion was observed by phalloidin staining. The chondrocytes were allowed to adhere more abundantly and uniformly on the silk microfibers than on the PCL microfibers (Figure 2C,D). Cell aggregation could even be induced in the silk microfibers. The high cell adhesion on the silk microfibers after a 24 h culture period was further confirmed by MTS assay (Figure 2E).

### 3.3. Cell Growth and Proliferation

**Figure 3:**
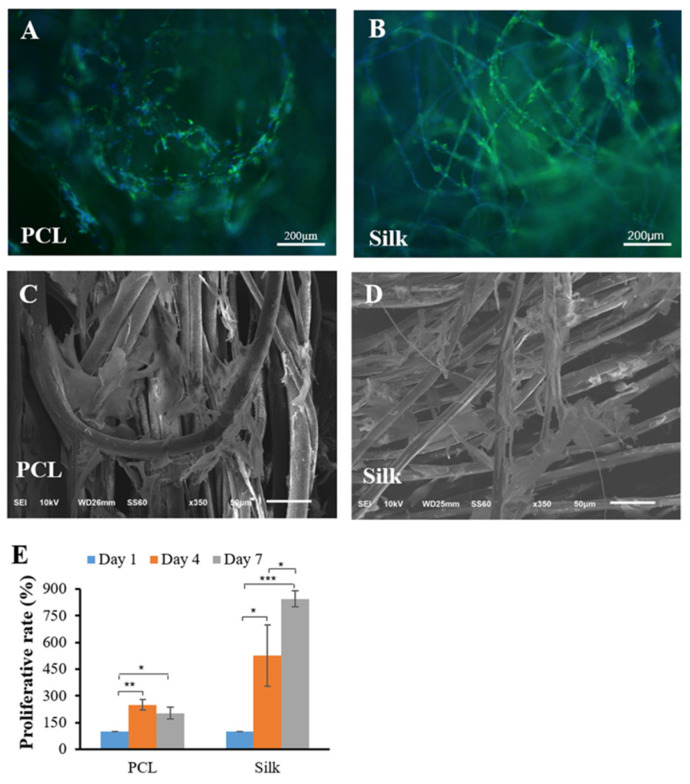
Fluorescent phalloidin and SEM images of both cell/silk and cell/PCL constructs were analyzed after 7 days of culture in growth medium. Fluorescent images of both constructs (**A**,**B**). SEM images of both constructs (**C**,**D**). MTS assay for proliferative rate of the cells on both microfibers (**E**). * *p* < 0.05, ** *p* < 0.01, and *** *p* < 0.001 relative to Day 1.

Both cell-laden microfibers were cultured for 7 days in the growth medium. Both constructs were shown to grow actively for up to 7 days (Figure 3A,B). After 7 days of culture, SEM images of chondrocytes grown on both microfibers were taken; these are shown in Figure 3C,D. The cells tightly attached themselves to a single microfiber, largely filled the spaces of the microfibers, and were highly flattened. The cell proliferation rate during the 7 days of culture was evaluated by MTS assay (Figure 3E). The silk microfibers showed an increasing growth rate for up to 7 days. The PCL microfibers exhibited increased cell proliferation on days 4 and 7 compared to day 1, although a slight decrease in growth was observed on day 7 relative to day 4. Particularly, the silk microfibers presented a more significant proliferation rate than the PCL microfibers with time.

### 3.4. Chondrogenic Phenotype Expression

**Figure 4:**
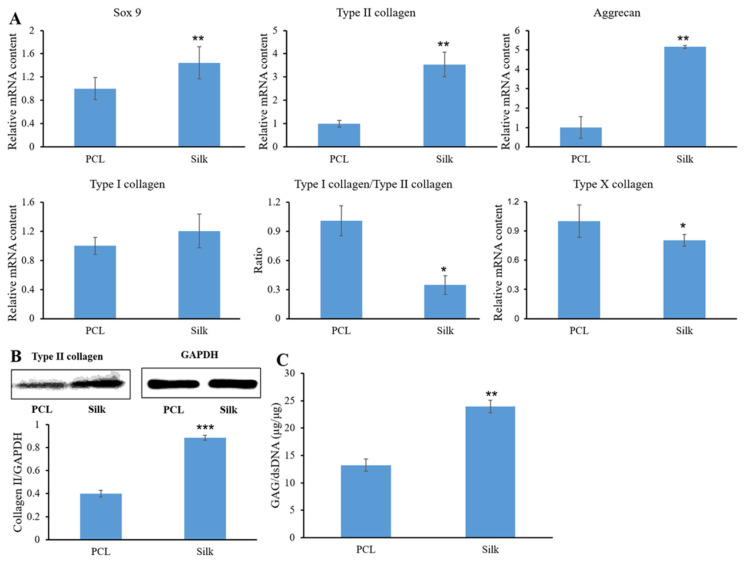
qPCR analysis of chondrogenic gene expression in cell-laden microfibers after 14 days of culture (**A**). * *p* < 0.05 and ** *p* < 0.01 relative to PCL. Representative Western blot image and quantification of type II collagen levels. Type II collagen expression was normalized to GAPDH (**B**). *** *p* < 0.001 vs. PCL. The DMMB assay was used to analyze GAG content in cell-laden microfibers after 14 days of culture. The GAG content was adjusted relative to the total DNA content of the constructs, showing a statistically significant difference (**C**). ** *p* < 0.01 vs. PCL.

Following a 14-day culture of cell-laden microfibers in chondrogenic medium, the expression levels of key chondrogenic genes (Sox9, type II collagen, and aggrecan), the dedifferentiation marker (type I collagen), and the hypertrophic chondrocyte marker (type X collagen) were assessed (Figure 4A). The results revealed that silk microfibers exhibited significantly higher expression of chondrogenic genes compared to PCL microfibers. Type I collagen expression did not significantly differ between the groups. The expression ratio of type I to type II collagen was markedly lower in silk microfibers containing cells than in PCL microfibers with cells. Type X collagen expression was lower in silk microfibers compared to PCL microfibers.

Western blot analysis (Figure 4B) revealed an enhanced expression of type II collagen protein in cell-laden silk microfibers compared to PCL microfibers. The DMMB assay was used to evaluate the extracellular matrix (ECM) composition of both cell-laden microfibers (Figure 4C). The findings revealed that the silk microfibers contained a greater amount of GAG products than the PCL microfibers.

## 4. Discussion

Through an evaluation of cell adhesion, proliferation, and phenotypic maintenance, silk microfibers were compared to PCL microfibers on rat chondrocytes. The results indicated that silk microfibers effectively promote chondrocyte adhesion and proliferation by upregulating the expression of cell adhesion-related proteins. Additionally, silk microfibers successfully preserve the phenotype of chondrocytes by upregulating chondrogenic genes, proteins, and extracellular matrix components, while downregulating the expression of hypertrophic chondrocyte markers.

Silk is widely used in clinical and research applications due to its excellent biocompatibility, adjustable biodegradability, and unique mechanical properties [15,16]. Silk has been widely recognized as a promising material for cartilage tissue engineering, as evidenced by several studies [17,18,19]. For example, Voga et al. demonstrated that canine adipose-derived stem cells cultured on silk films spontaneously differentiated into a chondrogenic lineage under non-specific culture conditions [20]. The authors suggested that this chondrogenic differentiation may be attributed to chondrocyte aggregation and diverse cell sources from different species. Talukdar et al. investigated the impact of varying cell seeding densities on the chondrogenic phenotype of bovine chondrocytes using silk scaffolds, revealing that higher cell densities enhanced the preservation of the chondrocyte phenotype [21]. Similarly, Kachi et al. showed that silk-decorated substrates facilitate chondrocyte aggregation and maintain the chondrogenic phenotype [22]. Thus, silk not only promotes cell growth and proliferation but also positively influences the maintenance of cell phenotypes. While the Arg-Gly-Asp (RGD) tripeptide sequence is recognized as a cell-binding site, Bombyx mori silk lacks this sequence, unlike non-mulberry silks [23]. In the current study, chondrocytes adhered more abundantly to silk microfibers than to PCL microfibers by day 1 post plating. The improved adhesion can be linked to the silk microfibers’ increased water affinity, which aids in cell attachment. Subsequently, the silk microfibers exhibited a significantly higher proliferative rate than the PCL constructs over the course of 7 days. Furthermore, these findings may also be linked to the N-terminal region of silk, which has been shown to promote cell growth [24].

Chondrogenesis during skeletal development begins with mesenchymal condensation, resulting in a cell mass which expresses cartilage-specific transcription factor Sox9 and differentiates into the chondrogenic lineage. Ultimately, the chondrocytes derived from this process undergo hypertrophic differentiation [25]. Therefore, chondrocyte aggregation in vitro is crucial for maintaining the chondrocyte phenotype [26]. In this study, chondrocytes exhibited significant proliferation on space-constrained scaffolds, with an extended culture duration enhancing cell density and aggregation, thus preserving the chondrocyte phenotype. Due to the particularly significant growth rate observed with the silk microfibers, chondrogenic-related markers (both at the gene and protein levels) were expressed at higher levels compared to those on the PCL microfibers. Silk also suppressed type X collagen expression, a marker linked to hypertrophic chondrocytes. In our previous study, we successfully restored the chondrocyte phenotype by culturing dedifferentiated chondrocytes in cell spheroids, further emphasizing the critical role of three-dimensional aggregation in preserving the chondrocyte phenotype [26]. When cells proliferate to fully cover the surface of microfibers, they begin to aggregate and grow in three dimensions, forming a microenvironment similar to that of cell spheroids. In the present study, due to the significantly enhanced proliferation rate of chondrocytes on silk microfibers compared to PCL microfibers, the three-dimensional aggregation of chondrocytes on silk microfibers was significantly superior. Therefore, silk microfibers demonstrate advantages over PCL microfibers in maintaining the chondrocyte phenotype. Silk may significantly contribute to cartilage repair, though the exact mechanisms need further study. Bhattacharjee et al. even developed chondroitin sulfate-conjugated silk to enhance GAG production through the MEK-ERK and p38-MAPK pathways [27].

Silk can be engineered into hydrogels, sponges, nanofibers, and microfibers for cartilage tissue applications [16,28]. These scaffolds are typically produced from processed regenerated silk solutions. However, achieving the desired fiber-type scaffolds through the electrospinning of regenerated silk solution can be challenging due to numerous complex parameters [29]. Lister et al. were the first to introduce natural silk sutures into clinical surgery [30]. At the time, one of the main concerns was the inflammatory reactions triggered by the presence of silk sericin. As a result, sericin was often removed from silk sutures to prevent adverse reactions. Altman et al. successfully utilized sericin-free silk to prepare artificial anterior cruciate ligaments that supported adult stem cell attachment, proliferation, and ligament-specific differentiation [31]. In the present study, sericin-free silk microfibers also demonstrated significant effects on chondrocyte proliferation and phenotype maintenance. Furthermore, the efficacy of fiber-type scaffolds has been confirmed in the treatment of osteoarthritis [32]. Thus, natural sericin-free silk microfibers are anticipated to become a promising biomaterial for repairing damaged cartilage in the future of orthopedic surgery and bioengineering.

## Figures and Tables

**Table 1:** Primer sequences of chondrogenic genes for qRT-PCR.

Gene	Forward Sequence	Reverse Sequence
*Sox9*	5′-CTGAAGGGCTACGACTGGAC-3′	5′-TACTGGTCTGCCAGCTTCCT-3′
*Type II collagen*	5′-GAGTGGAAGAGCGGAGACTACTG-3′	5′-CTCCATGTTGCAGAAGACTTTCA-3′
*Aggrecan*	5′-CTAGCTGCTTAGCAGGGATAACG-3′	5′-TGACCCGCAGAGTCACAAAG-3′
*Type I collagen*	5′-CGTGACCAAAAACCAAAAGT-3′	5′-GGGGTGGAGAAAGGAACAGA-3′
*Type X collagen*	5′-GATCATGGAGCTCACGGAAAA-3′	5′-CCGTTCGATTCCGCATTG-3′
*GAPDH*	5′-TGAACGGGAAGCTCACTGG-3′	5′-TCCACCACCCTGTTGCTGTA-3′

## Data Availability

The data generated in this study are presented in this paper.
